# Disseminated Histoplasmosis: Fighting a neglected killer of patients with advanced HIV disease in Latin America

**DOI:** 10.1371/journal.ppat.1008449

**Published:** 2020-05-14

**Authors:** Mathieu Nacher, Pierre Couppié, Loic Epelboin, Félix Djossou, Magalie Demar, Antoine Adenis

**Affiliations:** 1 CIC INSERM 1424, Centre Hospitalier de Cayenne, rue des Flamboyants, Cayenne French Guiana; 2 Dermatology Department, Centre Hospitalier de Cayenne, rue des Flamboyants, Cayenne French Guiana; 3 Infectious Diseases Department, Centre Hospitalier de Cayenne, rue des Flamboyants, Cayenne French Guiana; 4 Parasitology-Mycology laboratory, Centre Hospitalier de Cayenne, rue des Flamboyants, Cayenne French Guiana; 5 UMR Tropical Biome and Immuno-pathophysiology, Université de Guyane, Cayenne French Guiana; Vallabhbhai Patel Chest Institute, INDIA

## And then came HIV…

Histoplasmosis was first described in Panama in 1906 in a patient presenting miliary tuberculosis-like symptoms. [1] It is caused by the dimorphic fungi *Histoplasma* spp. *Histoplasma* is largely present in the Americas but has been described in Africa; Europe; South, Southeast, and East Asia; and Australia. [2] The mycelial phase grows in guano-enriched soils, and aerosolized infectious microconidia can be inhaled. [3] In the host, mycelia transform into yeasts, which usually cause mostly subclinical or a mild spontaneously resolving flu-like respiratory illness. However, when the inoculum is massive it can lead to severe and potentially fatal acute infections. A small proportion of patients with underlying lung disease, may develop chronic fibrotic apical lung infiltrates and cavitation. In immunosuppressed persons, *Histoplasma* progressively spread to other organs causing a disseminated infection, which, when left untreated, is mostly fatal. [4]

The HIV/AIDS pandemic increased the number of immunocompromized persons, and, in endemic areas, disseminated presentations of histoplasmosis became more frequent. They are clinically very similar to miliary and/or extrapulmonary tuberculosis, and differentiating between the two is thus difficult. [5] In addition, histoplasmosis–tuberculosis coinfections are relatively frequent. Serology is insufficiently sensitive in immunocompromized patients, and fungal culture is slow and may exceed one month. Depending on the level of immune suppression, the dissemination and severity of disseminated histoplasmosis will increase. [3] About 20% of disseminated forms are severe and require prompt diagnosis and treatment because they have high case fatality, often within days. Progressive disseminated histoplasmosis became an AIDS-defining infection in 1986. In the United States of America, where the presence of endemic histoplasmosis is well known, antigen detection tests, which allow to obtain rapid results, became available for clinicians in the late 1980s and liposomal amphotericin became available in 1998. [6,7] Thus, in the USA, there were rapid gains in identifying and treating disseminated histoplasmosis.

## The histoplasmosis iceberg in Latin American countries

However, in endemic areas outside of the USA, these gains were mostly not available. HIV/AIDS strategic plans in Latin America have largely been unaware of the existence and magnitude of the problem that was killing many of their patients. [8] Rare reports trickling from rare centers of excellence documented the frequency of cases of disseminated histoplasmosis in HIV-infected patients. Hence, in Panama, 7.6% of patients had culture-proven histoplasmosis [9]; in Venezuela, histoplasmosis was documented in 43 of 200 (21.3%) [10]; in Fortaleza, Brazil, of 378 consecutive hospitalized HIV patients, 164 (43.4%) had disseminated histoplasmosis [11]; A recent screening study in hospitalized HIV patients in Brazil found high *Histoplasma* antigen prevalence, ranging from 8.8% in Porto Alegre to 44.8% in Natal. [12] In French Guiana, with an overall incidence of 1.5 per 100 person years and 10 per 100 person years for patients with cluster of differentiation lymphocytes 4 (CD4) counts less than 50 per mm^3^, disseminated histoplasmosis has been the first AIDS-defining illness and the first cause of death in French Guiana; [13,14] among hospitalized HIV-infected patients with advanced HIV, 42% had disseminated histoplasmosis, among those with CD4 counts less than 50 per mm^3^, 85% had histoplasmosis. [15] With increased diagnostic sensitivity, numbers of diagnoses increase and deaths decrease. [16] In Guatemala, implementing antigen detection led to an increased number of annual diagnoses and a reduction of mortality in HIV-infected patients. [17] In Colombia, increasing awareness through training and antigen detection increased the number of diagnoses six-fold. [18] However, these data originate from histoplasmosis-aware centers, and in most hospitals across Latin America, histoplasmosis is not diagnosed and not treated, often being mistaken for tuberculosis, thereby leading to unnecessary deaths and inflated tuberculosis statistics.

Country by country estimates of the burden of disseminated histoplasmosis relative to HIV-associated tuberculosis showed that in 9 of 21 (43%) Latin American countries, the incidence of symptomatic disseminated histoplasmosis was greater than that of tuberculosis; furthermore, in 14 of 21 (67%) Latin American countries, the case fatality from disseminated histoplasmosis was higher than for tuberculosis, a situation overlooked by most national strategic plans. [19] Hence, nearly 40 years after the description of AIDS, disseminated histoplasmosis is still killing many patients. Despite testing and antiretrovirals, over 25% of those diagnosed with HIV have advanced disease; therefore, a significant number of persons are at risk for *Histoplasma* dissemination. In HIV-negative cases, therapeutic immunosuppression is also an increasing provider of disseminated cases. [20]

## The absence of antigen detection tests: A major laboratory diagnostic gap explaining neglect in low- and middle-income countries

Given the amazing successes in the fight against HIV, it seems surprising that this major cause of death has remained mostly unrecognized. Disseminated histoplasmosis is difficult to diagnose. Medical mycology is a rare specialty, and fungal culture requires special laboratories (biosafety levels 2 and 3) and takes weeks to grow. Serology is not sensitive for immunocompromized patients. Molecular diagnosis is now available in many high-income laboratories, but the required expertise and resources are still out of reach for many hospitals. This emphasizes the potential importance of antigen detection, for which enzyme immunoassays (EIAs) have been available for nearly 30 years but only in the USA. EIAs are rapid diagnostic methods that do not require special expertise or equipment and should thus be usable in most low-resource hospitals. [21] But if there is no data, there is no problem and then no need to collect data, a vicious circle that hampers the rise of awareness. [8]

## Therapeutic limitations in low- and middle-income Latin American countries

Treatment of severe disseminated histoplasmosis ideally requires liposomal amphotericin B induction, an expensive drug that is not available in most low- and middle-income endemic countries. Those who are diagnosed are given deoxycholate amphotericin B, an effective but toxic drug. For less severe patients, or after induction, itraconazole is given, and, while more widely available, it is not available everywhere. There again, the lack of awareness of the local epidemiology has significant consequences on country prioritization of diagnostic and therapeutic priorities.

## Awareness and capacity building in Latin America: 100 by 2025

Given this situation, for the past decade, the isolated researchers aware of the problem have gradually joined into a growing network to share their experiences. Some of these Latin American experts are mycologists who, naturally, are histoplasmosis-aware, others are clinicians stumbling on histoplasmosis while looking for leishmaniasis in endemic areas, and others are epidemiologists counting AIDS deaths. [22] Given the distances and insufficient air travel connectivity, early encounters were difficult. They were hard to fund, but they were propelled by shared concerns and situation analysis embodied in advocacy papers arguing that histoplasmosis is a neglected disease. Increasing publication numbers helped the Global Action Fund for Fungal Infections (GAFFI) to make the case for the inclusion of itraconazole in the WHO essential drugs list (2017) and Histoplasma antigen detection tests in the WHO list of essential diagnostic tests (2019). In 2017, WHO’s advanced HIV disease guidelines mentioned histoplasmosis. [23] With time, gatherings increased in size and became focused on more specific targets. In 2019, the Pan American Health Organization(PAHO) and WHO funded the biggest meeting so far in Manaus with 25 countries represented. One of the deliverables was to assemble a guideline development group for the drafting of specific WHO HIV-associated disseminated histoplasmosis diagnosis and treatment guidelines. The bottom-up self-organization around a neglected topic now seems to have been caught up by top-down efforts to move forward that will hopefully officially make histoplasmosis a problem to be tackled in all endemic countries. The 100 by 2025 goal in the Manaus Declaration aims for all hospitals in endemic countries to be supplied with diagnostic tests and effective treatments by 2025. The WHO/PAHO guidelines and stewardship should help push the agenda forward with country authorities and negotiate reduced pricing for liposomal amphotericin B.

## Histoplasmosis may also be killing numerous patients beyond Latin America

As we begin to unveil the burden of disease in Latin America, this is a signal for manufacturers of diagnostic tests that there is a potential market in low- and middle-income countries, and, hopefully, this will drive innovation to obtain cheap, reliable, and easy to use tests, ideally point-of-care tests. Specific efforts should aim to provide these tests in low and medium resource countries. *Histoplasma capsulatum* is known to be present throughout the world, and studies are starting to find it in significant numbers among HIV-infected patients. Hence, there are now reports of HIV-associated histoplasmosis in Cameroon, Nigeria, Ivory Coast, Congo, South Africa, India, China, and Southeast Asia.[24–26] In a recent study among 100 imported cases of histoplasmosis diagnosed between 2007 and 2018 in France, 55 were acquired in West and Central Africa. [27]

It is too early to estimate a precise burden, but given the number of immunocompromized patients in these regions, this may only be the tip of another iceberg and could amount to a considerable amount of morbidity and, in the absence of awareness, diagnosis, and treatment, deaths, as shown in Table 1.

**Table 1:** Simulations applying the mean incidence rate, the 10th percentile, and the 90th percentile for Latin America to the number of persons living with HIV in selected countries in Asia and Africa.

Country	HIV (Number)	Mean Histoplasmosis Incidence (1.48 per 100 Person Years)	Number Symptomatic (50%)^1^	Number of Deaths (Case Fatality = 40%)^1^	10th Percentile Histoplasmosis Incidence (0.66 per 100 Person Years)	Number Symptomatic (50%)^1^	Number of Deaths (Case Fatality = 40%)^1^	Incidence (2.9 per 100 Person Years)	Number Symptomatic (50%)^1^	Number of Deaths (Case Fatality = 40%)^1^	AIDS Deaths	Proportion of Aids Deaths (Mean)	Proportion AIDS Deaths 10th Percentile	Proportion AIDS Deaths 90th Percentile
South Africa	8000000	118400	59200	23680	52800	26400	10560	232000	116000	46400	87000	27.22	12.14	53.33
Ivory Coast	460000	6808	3404	1361	3036	1518	607	13340	6670	2668	19000	7.17	3.20	14.04
Cameroon	540000	7992	3996	1598	3564	1782	712	15660	7830	3132	17000	9.40	4.19	18.42
Nigeria	1900000	28120	14060	5624	12540	6270	2508	55100	27550	11020	66000	8.52	3.80	16.70
Indonesia	640000	9472	4736	1894	4224	2112	844	18560	9280	3712	41000	4.62	2.06	9.05
Thailand	480000	7104	3552	1420	3168	1584	633	13920	6960	2784	26000	5.46	2.44	10.71
Myanmar	240000	3552	1776	710	1584	792	316	6960	3480	1392	11000	6.46	2.88	12.65
India	2110000	31228	15614	6245	13926	6963	2785	61190	30595	12238	62000	10.07	4.49	19.74
China (low estimate)	430000	6364	3182	1272	2838	1419	567	12470	6235	2494	9333	13.64	6.08	26.72
Total	14800000	219040	109520	43808	97680	48840	19536	429200	214600	85840	338333	12.95	5.77	25.37

^1^Considering that 50% of incident cases would be symptomatic and that 40% of the symptomatic would die.

Hence, factoring the number of persons infected with HIV in 2018 in a selection of African and Asian countries by the average and 10th and 90th percentiles of the Latin American incidence rate, with a 50% symptomatic proportion and a conservative 40% mortality, suggests, for these countries, nearly 110,000 [48,000–430,000] annual cases and nearly 44,000 [20,000–85,000] deaths. This very crude and very criticizable estimate could be biased by differences in environmental conditions, host, and pathogen genetic backgrounds on different continents. It should nevertheless stimulate research documenting the true burden of histoplasmosis among persons living with HIV throughout the world and the natural history of dissemination as immunosuppression gradually deepens, which could lead to preemptive treatment before severe symptoms appear. In conclusion, over 100 years after the first description of histoplasmosis in a patient presenting tuberculosis-like symptoms the diagnostic difficulty remains for most clinicians across the world, with lethal consequences.
